# Cysteine Inhibits the Growth of Fusarium oxysporum and Promotes T-2 Toxin Synthesis through the Gtr/Tap42 Pathway

**DOI:** 10.1128/spectrum.03682-22

**Published:** 2022-10-31

**Authors:** Mei Qiu, Yijia Deng, Qi Deng, Lijun Sun, Zhijia Fang, Yaling Wang, Xiaoyue Huang, Jian Zhao

**Affiliations:** a College of Food Science and Technology, Guangdong Ocean University, Zhanjiang, China; b Guangdong Provincial Key Laboratory of Aquatic Product Processing and Safety, Zhanjiang, China; c Guangdong Provincial Engineering Technology Research Center of Marine Food, Zhanjiang, China; d Key Laboratory of Advanced Processing of Aquatic Products of Guangdong Higher Education Institution, Zhanjiang, China; e School of Chemical Engineering, The University of New South Wales, Sydney, New South Wales, Australia; Universidade de Sao Paulo

**Keywords:** amino acid, T-2 toxin, target of rapamycin complex 1 (TORC1) pathway

## Abstract

Fusarium oxysporum is ubiquitous and can easily contaminate food during processing and storage, potentially producing T-2 toxin, which can pose a severe health risk to public health. Previous research on the presence of T-2 has focused on starch-rich foods, while protein- and amino acid-rich foods have received relatively little attention. In this study, the effects of amino acids on the growth of F. oxysporum and its T-2 production were investigated by gene deletion and complementation experiments. The results showed that amino acids, including aspartic acid, methionine, isoleucine, serine, phenylalanine, and cysteine, significantly inhibited the growth of F. oxysporum, while promoting T-2 synthesis, with cysteine having the most pronounced effect. The target of rapamycin complex 1 (TORC1) is a key pathway in response to a variety of amino acids, including cysteine. *gtr2* and *tap42* were found to be negative regulators of T-2 synthesis. The study highlights the elevated risk of T-2 production by F. oxysporum in cysteine-rich foods and the need to take appropriate measures to prevent and control the potential harm that such foods may present to public health.

**IMPORTANCE** F. oxysporum and its T-2 contamination of food not only leads to food wastage but also poses a major food safety challenge to humans. The growth and T-2 production characteristics of F. oxysporum in high-protein substrates are considerably different from those in grains. Here, we show that the abundant free amino acids in a protein-rich food matrix are a key regulatory factor for the growth of, and toxin production by, F. oxysporum. Cysteine has the most pronounced effect on inhibiting mycelial growth and promoting T-2 synthesis through the TORC1 pathway. This implies that consumers tend to overlook T-2 contamination due to the poor growth of F. oxysporum in food rich in protein and amino acids, especially cysteine. Therefore, particular attention should be paid to the protection of those products.

## INTRODUCTION

Fusarium spp. are ubiquitous in the environment and can easily contaminate food (1, 2). Usually, Fusarium spp. exhibit particularly high contamination rates in crops such as oats, corn, and rice, as the organism prefers carbohydrate-rich substrates (3). However, a few articles have reported that Fusarium also easily contaminates high-protein substrates, such as dry-cured ham and dried fish (2, 4, 5). Several species of fungi of the genus Fusarium produce T-2 toxin (T-2), which is one of the most potent mycotoxins and can cause acute and chronic illnesses in humans (3). In our investigation, a T-2-producing Fusarium strain was first isolated from dried fish and identified as Fusarium oxysporum Fo17 by internal transcribed spacer (ITS) (6), translation elongation factor 1-alpha (TEF-1α), and β-tubulin (TUB) sequence analysis (see Table S1 and Fig. S1 in the supplemental material). This new discovery suggests that F. oxysporum, as a food spoilage and mycotoxigenic fungus, should be included in the new food hygiene monitoring target. Therefore, it is necessary to find an effective method to control the growth and T-2 synthesis of F. oxysporum in food.

Amino acids play an important role in many biological processes, including protein synthesis, cell growth, development, and secondary metabolism. Studies have shown that amino acids are closely related to fungal growth and mycotoxin production. For example, Jimenez et al. (7) showed that increasing amounts of free amino acids (FAA) can inhibit the growth of Fusarium fujikuroi but promote fumonisin B_1_ production. In Fusarium graminearum, the methionine synthesis gene *fgmetb* regulates spore germination, mycelial growth, deoxynivalenol production, and pathogenicity (8). Shiobara et al. (9) showed that glycine, serine, and threonine are the only nitrogen sources that inhibit trichothecene biosynthesis. However, it is unclear what role amino acid metabolism plays in regulating the growth and T-2 production of F. oxysporum and whether different amino acids play a similar or different role in the regulation.

**FIG 1:**
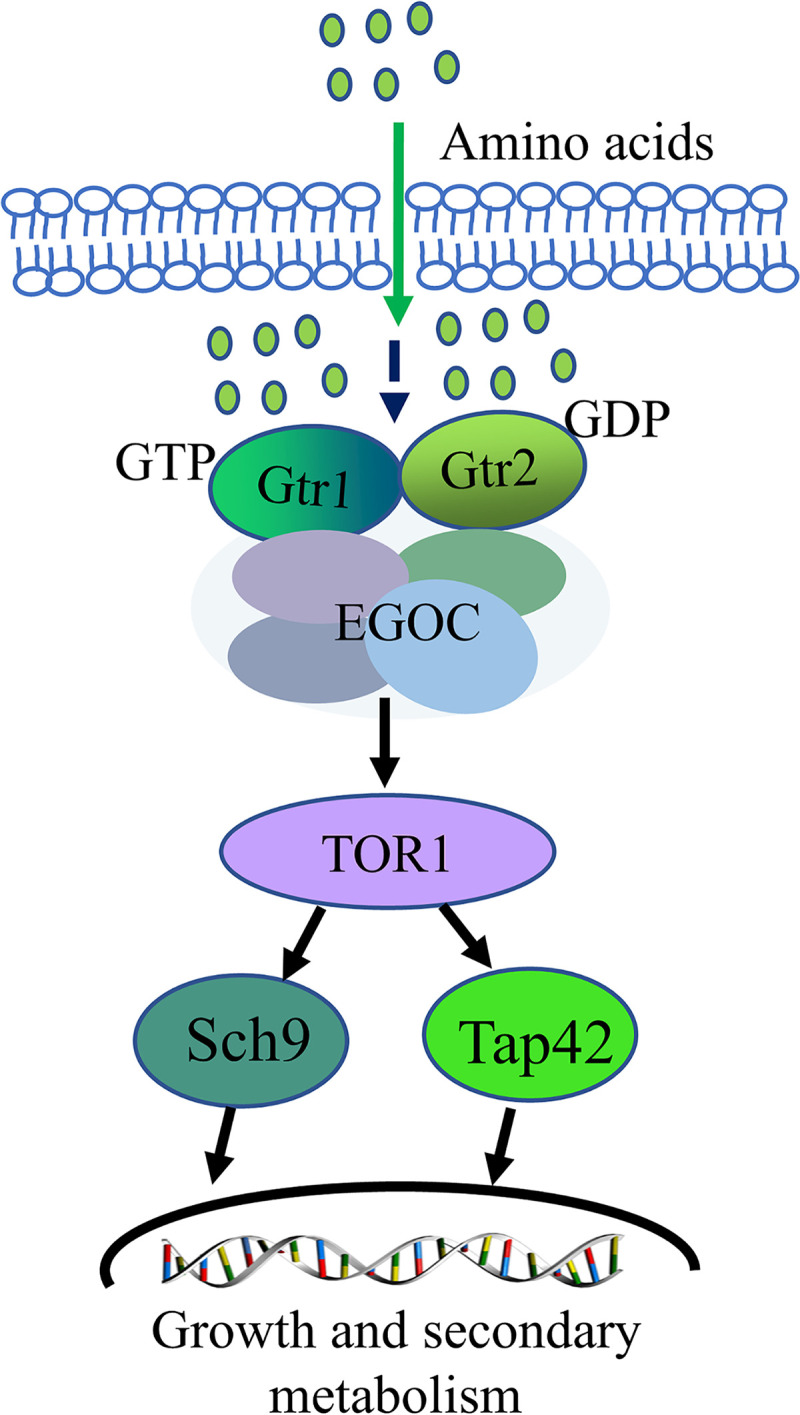
Model of the TORC1-mediated signaling pathway in S. cerevisiae (12). EGOC, EGO complex.

These processes of amino acid metabolism have been thoroughly studied in Saccharomyces cerevisiae (10–13). The target of rapamycin (TOR) signaling pathway, which is at the core of regulating growth and secondary metabolism in response to the nutrient supply in S. cerevisiae, is composed of two structurally distinct complexes: TOR complex 1 (TORC1) and TOR complex 2 (TORC2). In eukaryotes, nitrogen and amino acids have been identified as key modulators of TORC1 activity (10). Amino acids regulate cell growth and secondary metabolism by activating the Gtr1-Gtr2 complex, recruiting the TORC1 complex, and activating the Tap42 or Sch9 branches downstream of TORC1 (Fig. 1) (11–13). Specific amino acids, such as leucine, glutamine, asparagine, arginine, aspartic acid, methionine, and cysteine, are potent activators of S. cerevisiae TORC1 and Schizosaccharomyces pombe TORC1 signaling (14–18). Under favorable external amino acid nutritional conditions, the Gtr1-Gtr2 complex of S. cerevisiae directly interacts with key elements in TORC1 (19) and transmits signals to downstream Tap42 and Sch9 effector factors (20, 21).

The above studies indicated that the abundant FAA in a protein-rich food matrix were a key regulatory factor for the growth of, and toxin production by, F. oxysporum. Additionally, the roles of the key genes *gtr1*, *gtr2*, *tap42*, and *sch9* in the TORC1 pathway in response to amino acid regulation of growth and T-2 synthesis in F. oxysporum are considerably less known. Therefore, we used gene deletion and complementing techniques to explore how amino acids regulate the growth and T-2 biosynthesis of F. oxysporum. Our research makes an important contribution to the exploration of T-2 biosynthesis, amino acid metabolism, and TORC1 signaling regulations and lays a foundation for controlling the production of mycotoxins by F. oxysporum.

## RESULTS

### Cysteine significantly inhibited the growth of F. oxysporum and promoted T-2 synthesis.

**FIG 2:**
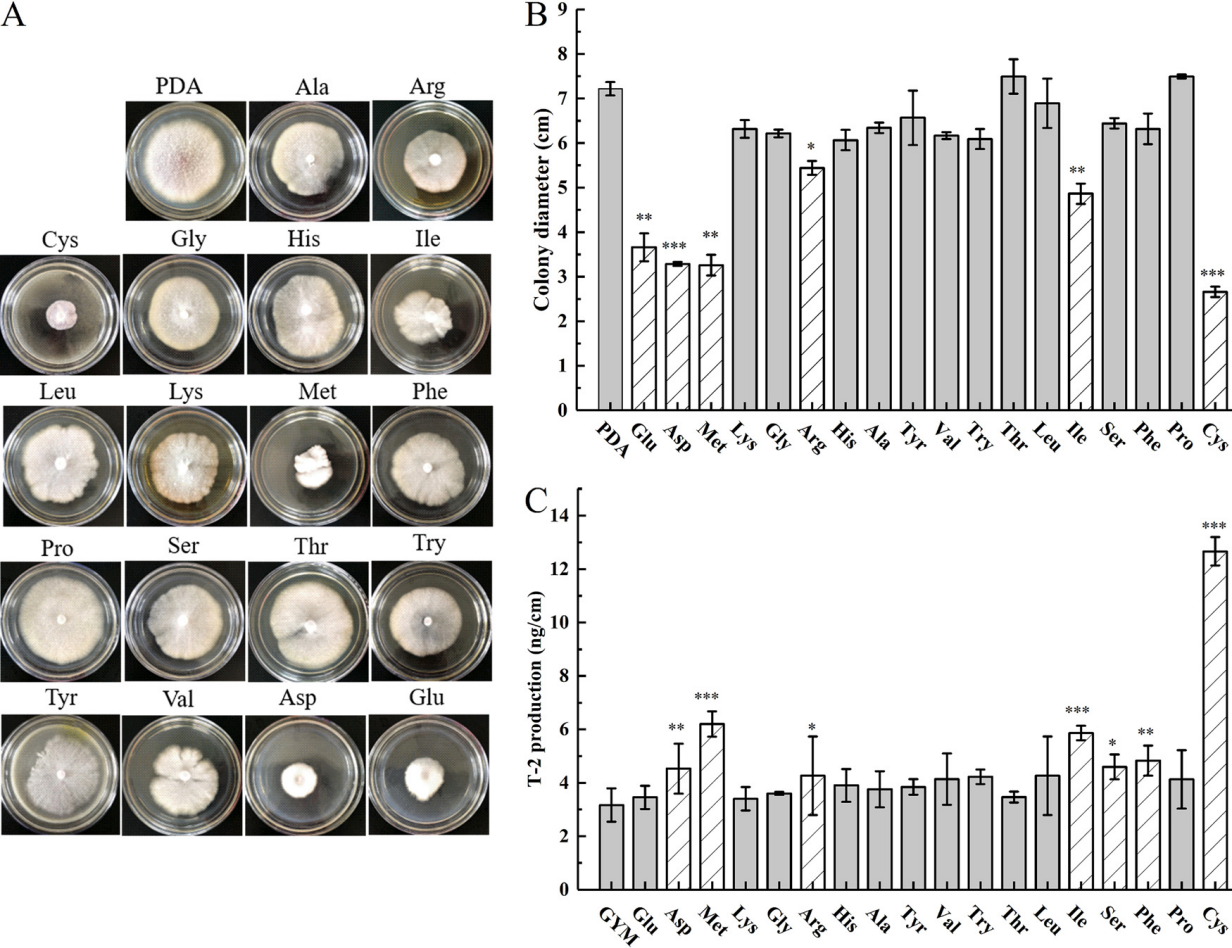
Effect of different amino acids on the growth and T-2 production of Fusarium oxysporum Fo17. (A) Colony morphology on PDA, (B) colony diameter on PDA, and (C) concentration of T-2 on GYM. Both PDA and GYM were supplemented with 18 different amino acids (5 mg/mL). *, *P < *0.05; **, *P < *0.01; ***, *P < *0.001.

Supplementation of potato dextrose agar (PDA) with amino acids had a significant impact on mycelial growth (Fig. 2). When each of the 18 amino acids was added to the PDA medium, the mycelial growth of F. oxysporum was irregular and slow. Radial growth was reduced with all of 18 amino acids, except proline and threonine. In particular, glutamic acid, aspartic acid, methionine, arginine, isoleucine, and cysteine significantly inhibited the growth of fungal colonies, with cysteine exhibiting the most pronounced inhibitory effect (Fig. 2A and B).

Analysis of T-2 in glucose yeast medium (GYM) showed that the concentration of the mycotoxin was higher in all the amino acid-supplemented media, demonstrating that all 18 amino acids promoted T-2 production. However, media supplemented with aspartic acid, methionine, arginine, isoleucine, serine, and phenylalanine had significantly larger amounts of T-2. In particular, cysteine-supplemented GYM accumulated the highest concentration of T-2, more than 4 times higher than that in GYM without amino acid supplementation (Fig. 2C). These results demonstrate that amino acids significantly inhibited the growth and promoted the T-2 biosynthesis of F. oxysporum, with cysteine exhibiting the strongest effects.

### Deletion and complementation of key genes responsive to amino acids in the TORC1 pathway.

**FIG 3:**
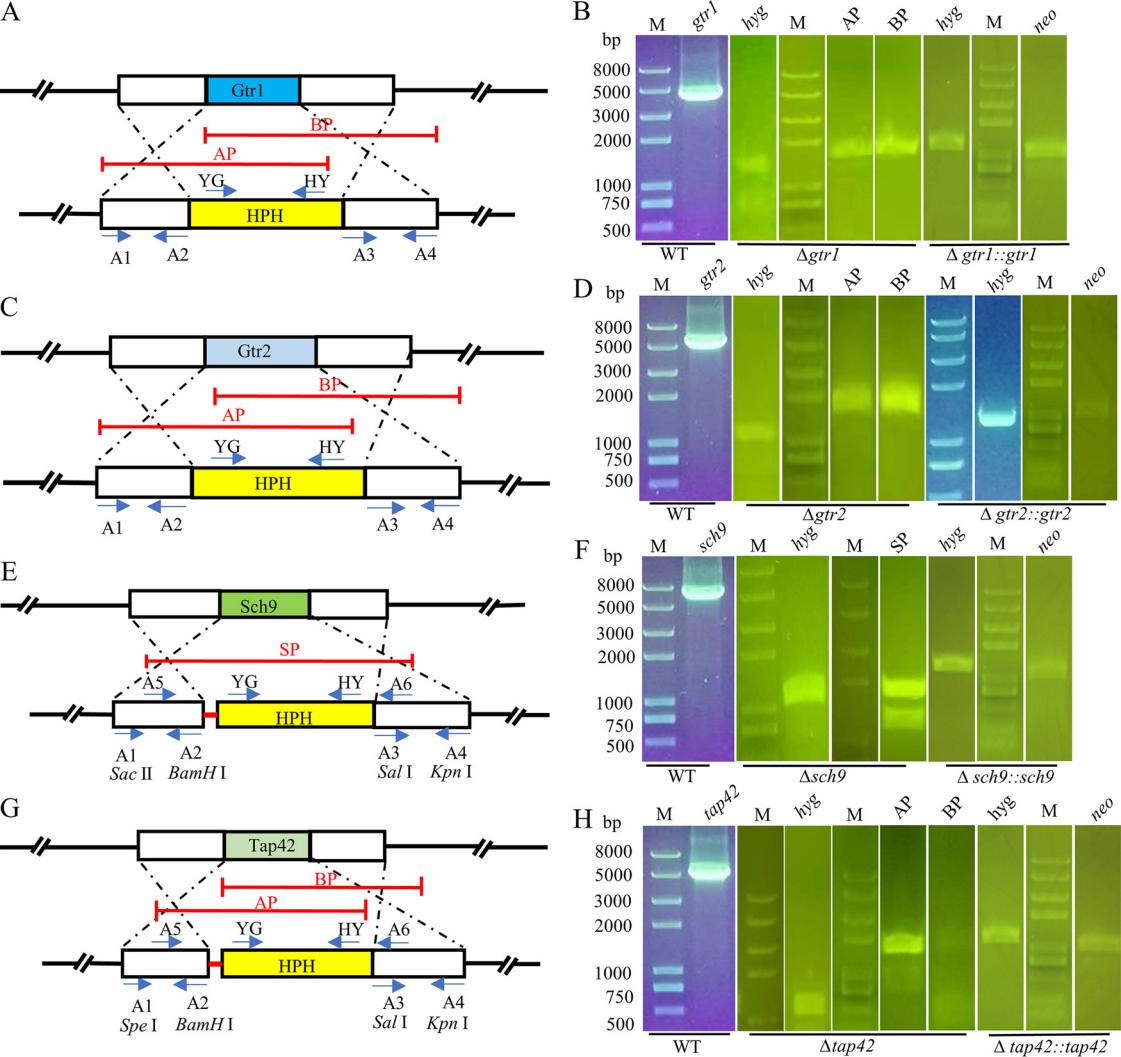
Schematic representation of the *gtr1*, *gtr2*, *sch9*, and *tap42* disruption strategy. (A, C, E, and G) Construction strategy for the Δ*gtr1*, Δ*gtr2*, Δ*sch9*, and Δ*tap42* strains, respectively, using the hygromycin resistance gene. (B, D, F, and H) PCR was used to confirm the gene deletion and complemented strains for the *gtr1*, *gtr2*, *sch9*, and *tap42* strains, respectively. M, DNA markers; *hyg*, PCR production of the hygromycin resistance gene; AP, fusion fragment of the *gtr1*, *gtr2*, and *tap42* upstream fragment and YG. BP, fusion fragment of the *gtr1*, *gtr2*, and *tap42* downstream fragment and HY; SP, PCR was conducted to confirm the *sch9* gene deletion mutants; *neo*, the *neo* gene for gene complement verification.

The strategy for construction of Δ*gtr1*, Δ*gtr2*, Δ*tap42*, and Δ *sch9* is illustrated in Fig. 3A, C, E, and G. The hygromycin resistance gene *hyg* was used to identify the positive mutant strains. A PCR test was then used to confirm the transformants, and the results demonstrated that both the AP and BP or SP fragments were present in the Δ*gtr1*, Δ*gtr2*, Δ*tap42*, and Δ *sch9* deletion mutants (Fig. 3B, D, F, and H), revealing that the gene deletion mutants Δ*gtr1*, Δ*gtr2*, Δ*sch9*, and Δ*tap42* were generated by homologous recombination of *gtr1*, *gtr2*, *sch9*, and *tap42*, respectively. Furthermore, a sequencing analysis was conducted to confirm the deletion mutants (see Fig. S2 to S8 in the supplemental material). The *hyg* and *neo* fragments in the complemented transformants demonstrated that the target gene is ectopically integrated into the genome of the corresponding complemented strain (Fig. 3B, D, F, and H).

### The TORC1 pathway is involved in F. oxysporum colony morphology and T-2 synthesis.

**FIG 4:**
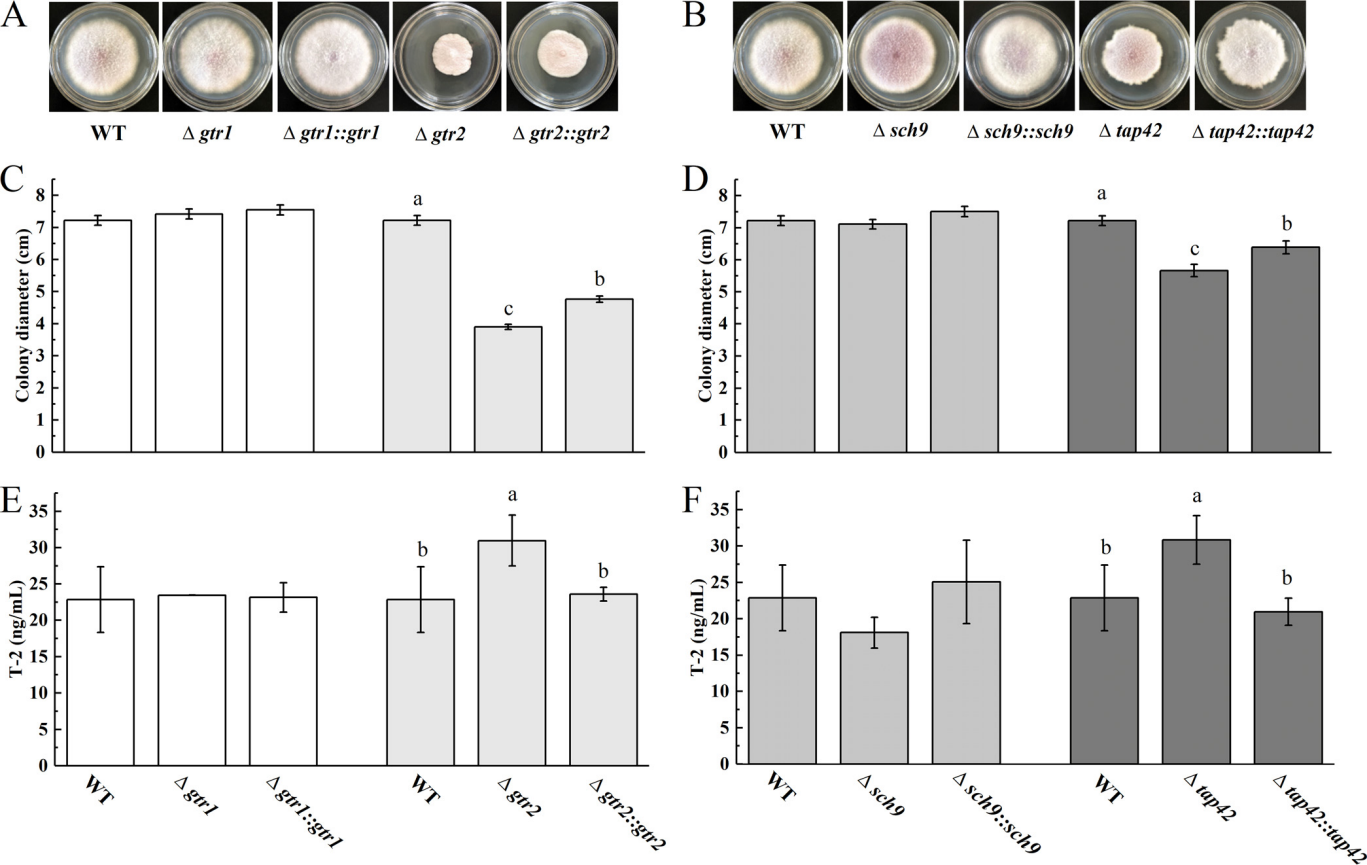
Δ*gtr1*, Δ*gtr2*, Δ*sch9*, and Δ*tap42* differed in their impact on mycelial growth and T-2 production. (A, B) Colony phenotypes of the indicated strains after being grown on PDA for 7 days. (C, D) Statistical analysis of mycelial growth on PDA for 7 days. (E, F) Statistical analysis of T-2 production on GYM for 14 days. Different letters above the bars represent significantly different values (*P < *0.05).

When wild-type (WT) strains, deleted mutants, and complementation mutants were cultured on PDA at 28°C for 7 days, the deleted mutants had higher pigmentation, and Δ*gtr1* and Δ*sch9* had marginally decreased radial growth compared with the WT and complemented strains (Fig. 4). Compared to WT, the mycelial growth of the Δ*gtr2* and Δ*tap42* mutants was significantly smaller and slower. Furthermore, morphological defects in the Δ*gtr2*::*gtr2* and Δ*tap42*::*tap42* mutants were partially recovered (Fig. 4A, B, C, and D). These data indicate that *gtr2* and *tap42* played an important role in the colony morphology of F. oxysporum. In addition to the relatively small changes in mycelial growth, the T-2 production in the Δ*gtr1* and Δ*sch9* mutants was also only slightly different from that of the WT. In contrast, the T-2 production of the Δ*gtr2* and Δ*tap42* mutants was significantly higher than that of the WT and complemented mutants. Importantly, the capacity for T-2 biosynthesis in the Δ*gtr2*::*gtr2* and Δ*tap42*::*tap42* mutants was restored to the WT level (Fig. 4E, F), indicating that the alteration in T-2 production by the Δ*gtr2* and Δ*tap42* mutants was directly caused by the destruction of the *gtr2* and *tap42* genes in F. oxysporum. Thus, *gtr2* and *tap42* are negative regulators of T-2 synthesis in F. oxysporum. In summary, the results revealed that *gtr2* and *tap42* in the TORC1 pathway regulate F. oxysporum mycelial growth and T-2 synthesis.

### Cysteine regulates the growth and T-2 synthesis of F. oxysporum through the TORC1 pathway.

**FIG 5:**
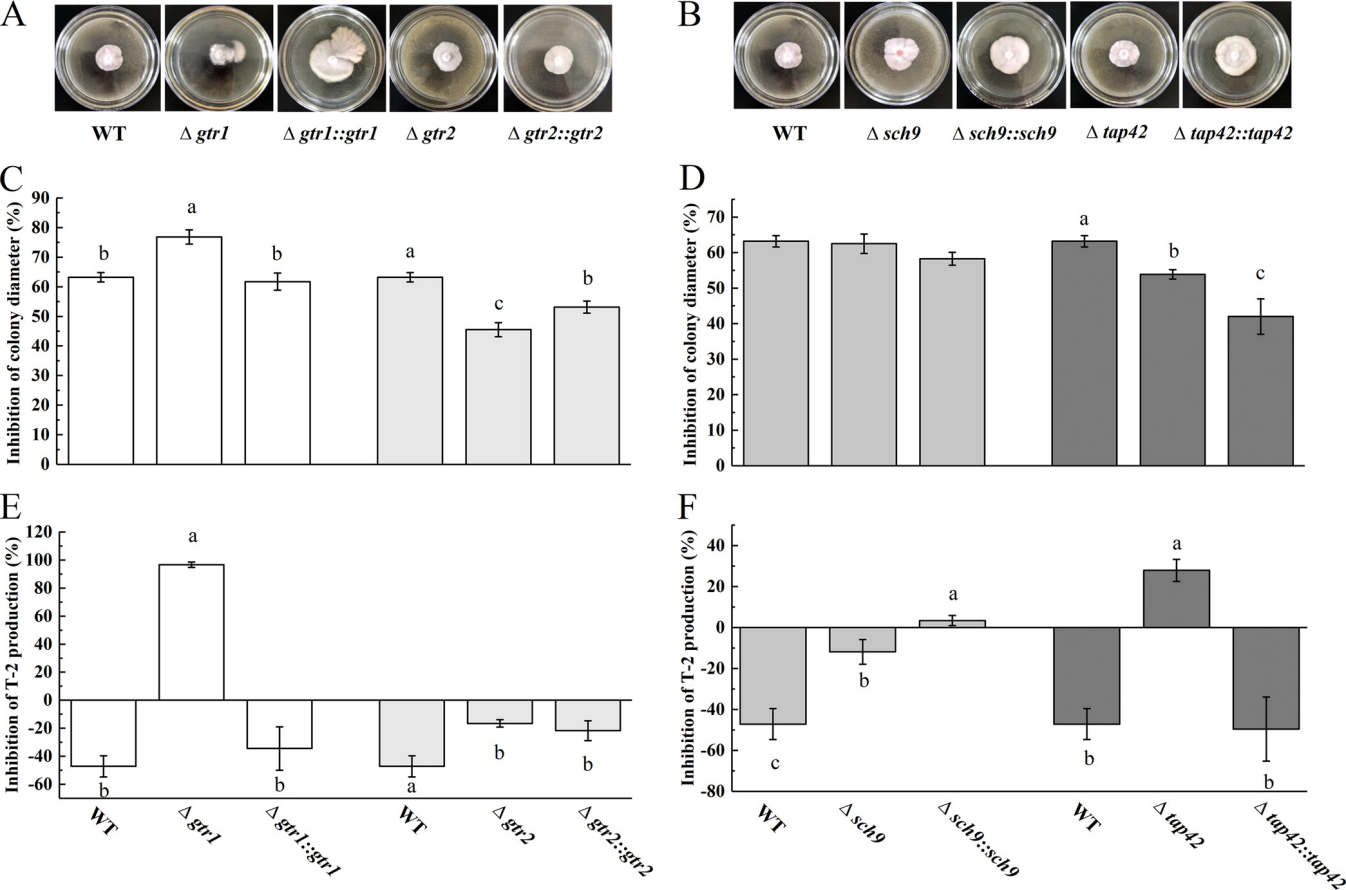
Response of the WT, Δ*gtr1*, Δ*gtr2*, Δ*sch9*, and Δ*tap42* and Δ*gtr1*::*gtr1*, Δ*gtr2*::*gtr2*, Δ*sch9*::*sch9*, and Δ*tap42*::*tap42* strains to 5 mg/mL cysteine. (A, B) Morphology of the indicated strains grown on PDA supplemented with 5 mg/mL cysteine for 7 days; (C, D) inhibition rate of radial mycelial growth of the strains; (E, F) inhibition rate of T-2 production of the strains cultured for 14 days in liquid GYM supplemented with 5 mg/mL cysteine. The inhibition rate in panels C to F was obtained by comparison with the wild type, grown on PDA or in GYM without cysteine supplementation. Different letters above the bars represent significantly different values (*P < *0.05).

When the WT strains, deleted mutants, and complemented mutants were grown on PDA supplemented with 5 mg/mL cysteine, the results showed that the growth of the Δ*gtr1* mutant decreased, that of the Δ*gtr2* and Δ*tap42* mutants increased, and that of the Δ*sch9* mutant did not change (Fig. 5A, B, C, and D). These results indicated that *gtr1* responded to cysteine to promote mycelial growth, while *gtr2* and *tap42* responded to cysteine to inhibit mycelial growth, and *sch9* was not a key gene in this process. Furthermore, compared with the WT strain, T-2 biosynthesis was severely blocked in the *gtr1* and *tap42* strains in GYM supplemented with cysteine (*P < *0.05), and T-2 biosynthesis in the Δ*gtr2* and Δ*sch9* strains was also reduced. However, compared with WT strains without cysteine, *gtr2* and *sch9* promoted T-2 synthesis (Fig. 5E and F), indicating that these two mutants were not involved in cysteine regulation of T-2 synthesis in F. oxysporum. Thus, cysteine inhibited the mycelial growth of F. oxysporum through the Gtr2/Tap42 pathway, while promoting T-2 synthesis through the Gtr1/Tap42 pathway.

## DISCUSSION

The effects of amino acids on the growth and T-2 production of F. oxysporum were studied for the first time in this study. First, we found that amino acids inhibited mycelial growth, and the effect of the different amino acids on the growth of F. oxysporum was not the same. The influence of FAA on F. oxysporum was different from their influence on Aspergillus spp., as indicated by the results of Wang et al. (22), who reported that there was almost no difference in the growth state of Aspergillus flavus when amino acids such as aspartic acid, glutamate, proline, and alanine were added to Czapek Dox medium. In addition, we found that amino acids in the growth medium also impacted T-2 biosynthesis. Most of the amino acids studied promoted T-2 synthesis, but the effect was especially pronounced with cysteine. Previously, it was reported that fungicides and preservatives inhibited fungal growth, while stimulating mycotoxin synthesis. For example, the application of suboptimal concentrations of strobilurins enhanced the biosynthesis of mycotoxins (23). Also, studies on Penicillium verrucosum have shown that at suboptimal concentrations, preservatives such as calcium propionate and potassium sorbate inhibit mycelial growth but increase ochratoxin A biosynthesis (24, 25). These studies revealed that unfavorable nutritional conditions enhance the toxigenic potential of fungi. In this study, for the first time, we found that amino acids had similar effects on the growth and T-2 synthesis of F. oxysporum. In particular, cysteine induced the lowest growth rate and the highest T-2 accumulation by F. oxysporum.

How does F. oxysporum respond to cysteine to regulate growth and T-2 synthesis? It is known that TORC1 is one of the main signaling pathways that regulate fungal growth and toxicity (7, 12). Previous studies have reported that FgGtr1, FgGtr2, and FgSch9 regulate the growth of aerial mycelia and deoxynivalenol synthesis in Fusarium graminearum (26). Through gene deletion and complementation experiments, we found that Gtr2/Tap42 were key genes regulating the growth and T-2 synthesis of F. oxysporum, suggesting that there are interspecies differences in the key genes regulating growth and secondary metabolism in the TORC1 pathway. Furthermore, the Δ*gtr2* mutant grew less well than the other deletion mutants, indicating that *gtr2* plays a dominant role in F. oxysporum growth, confirming the report by Binda et al. (19). However, in the presence of cysteine, synthesis of T-2 by the Δ*gtr2* mutant decreased significantly, indicating that *gtr1* and *gtr2* perform different physiological functions in response to specific cysteine signals. It has been reported that TORC1 activates the Sch9 kinase signaling branch and represses the Tap42-PP2A phosphatase branch required for the amino acid synthesis and nitrogen assimilation pathways when nutrients are abundant (27). On the contrary, in this study, we found that cysteine inhibited mycelial growth and promoted T-2 synthesis through Tap42 signaling, which may be due to the fact that cysteine contains highly reactive sulfhydryl groups that are not conducive to F. oxysporum growth and can trigger a state transition in TORC1 activity. This downregulates *sch9* and activates the Tap42-PP2A branch and the associated responses coping with cysteine limitation (28). Thus, this study demonstrates that F. oxysporum, and possibly other fungi, can produce more mycotoxins under amino acid-rich environmental stress. This implies that appropriate control measures should be taken to control F. oxysporum and its T-2 production in food rich in protein and amino acids, especially cysteine.

### Conclusion.

In this study, we found that amino acids can significantly impact the growth and T-2 production of F. oxysporum. Specifically, aspartic acid, methionine, arginine, isoleucine, serine, and cysteine inhibited the growth of F. oxysporum, while promoting T-2 synthesis, with cysteine having the most pronounced effect. Cysteine inhibited the mycelial growth of F. oxysporum through the Gtr2/Tap42 pathway and promoted T-2 synthesis through the Gtr1/Tap42 pathway. From the perspective of human and animal health, food rich in cysteine can be especially susceptible to the accumulation of T-2 by F. oxysporum, and particular attention should be paid to the protection of those products.

## MATERIALS AND METHODS

### Strains and culture conditions.

The microbial strains used in this study are listed in Table 1. Fungal cultures were maintained on potato dextrose agar (PDA) at 4°C. For the mycelial growth assay, colonies of the wild type (WT) and transformants on PDA were aseptically cut with a round corer (5 mm in dimeter), and the cored fungal culture was transferred onto PDA and PDA supplemented with 5 mg/mL amino acids (9). The cultures were incubated at 28°C for 7 days in the dark. Each experiment was repeated three times.

**TABLE 1:** Strains and plasmids used in this study

Strain or plasmid	Genotype or description
F. oxysporum Fo17^*a*^	Wild type
Δ*gtr1*	Δ*gtr1*::*hyg*
Δ*gtr2*	Δ*gtr2*::*hyg*
Δ*sch9*	Δ*sch9*::*hyg*
Δ*tap42*	Δ*tap42*::*hyg*
Δ*gtr1*-complemented	Δ*gtr1*::*gtr1*, *neo*, *hyg*
Δ*gtr2*-complemented	Δ*gtr2*::*gtr2*, *neo*, *hyg*
Δ*sch9*-complemented	Δ*sch9*::*sch9*, *neo*, *hyg*
Δ*tap42*-complemented	Δ*tap42*::*tap42*, *neo*, *hyg*
pClone007	Vector for blunt-end gene cloning
pBluescript KS(±) (pBS) −HPH1^*b*^	Vector for gene deletion
pCAMBIA1300-neo	Vector for gene complementation

a GDMCC accession number 60824.

b HPH1, hygromycin B phosphotransferase gene 1.

### Determination of T-2 toxin.

The production of T-2 toxin by the fungal strains was assessed in glucose yeast medium (GYM; 10 g glucose, 5 g yeast extract, 1.0 g NH_4_H_2_PO_4_, 0.2 g KCl, 0.2 g MgSO_4_·7H_2_O, 1 mL CuSO_4_·5H_2_O [5 mg/mL], 1 mL ZnSO_4_·7H_2_O [10 mg/mL]) and GYM supplemented with 5 mg/mL amino acids. Cores of all strains 5 mm in diameter were transferred into 5 mL GYM and GYM supplemented with 5 mg/mL amino acids, and the cultures were incubated in the dark at 28°C for 14 days at 120 rpm/min for T-2 production. After that, an equal volume of ethyl acetate was added to the GYM, followed by vertical oscillation for 10 min and centrifugation at 5,000 rpm/min for 5 min to collect 2.5 mL supernatant. The supernatant was evaporated with nitrogen at 60°C, redissolved with 1 mL 30% methanol, and filtered using a 0.22-μm filter. Liquid chromatography-tandem mass spectrometry (LC-MS/MS) was used to analyze the T-2 production as previously described (29).

### Gene deletion and complementation.

To determine the functions of *gtr1*, *gtr2*, *sch9*, and *tap42* in response to amino acids, constructs for deletion of these genes and complementation of F. oxysporum were created as described previously (26, 30). Gene knockout strains were constructed by substituting target genes in F. oxysporum with the hygromycin resistance gene in plasmid pBluescript KS(±) (pBS) −HPH1. The *gtr1*, *gtr2*, *tap42*, and *sch9* gene sequences were downloaded from the F. oxysporum gene database (http://www.broadinstitute.org/annotation/genome/fusarium_group/MultiHome.html). Double-joint fusion PCR was performed to create the deletion constructs. The purified PCR products were transformed into Fo17 protoplasts. Putative gene deletion mutants were identified by PCR assay with primers 11 to 32 (see Table S2 in the supplemental material) and were further confirmed by sequencing analysis.

For complementation, the target gene with its native promoter was amplified using primers 33 to 40 (Table S2) and then cloned into the vector pCAMBIA1300-neo. The recombinant plasmid, harboring a neomycin resistance marker, was transformed into protoplasts of the mutant. PCR analysis of the *neo* gene in the complemented vector pCAMBIA1300-neo was performed to identify the complemented strains.

### Statistical analysis.

Data are presented as the mean ± standard deviation. One-way analysis of variance (ANOVA) was used for multiple comparisons, followed by Tukey’s multiple-comparison test. Student’s *t* test was used to compare two means for differences. Statistical analyses were performed using SPSS v25, and differences were regarded as significant at *P* values of <0.05.

### Data availability.

F. oxysporum Fo17 has been deposited at the Guangdong Microbial Culture Collection Center, China (GDMCC), under the accession number GDMCC60824. The ITS, TEF-1α, and TUB sequences are available at GenBank under the accession numbers OP412776, OP330068, and OP382711, respectively.
