# Supplementary material for: Cysteine Inhibits the Growth of Fusarium oxysporum and Promotes T-2 Toxin Synthesis through the Gtr/Tap42 Pathway
Source: Microbiol Spectr. 2022 Oct 31;10(6):e03682-22. doi: 10.1128/spectrum.03682-22 (PMC9769839; doi:10.1128/spectrum.03682-22)
Supplement: Supplemental file 1 — Supplemental material. Download spectrum.03682-22-s0001.pdf, PDF file, 1.2 MB [file spectrum.03682-22-s0001.pdf]

Supplemental - Tables

TABLE S1 Primers, GenBank accession numbers and amplification procedures used for strain identification in this study

Sequence (accession)	Primer Sequences (5' to 3')	Amplification Program
ITS (OP412776)	ITS1: TCC GTA GGT GAA CCT GCG G<br>ITS4: TCC TCC GCT TAT TGA TAT GC	Started with an initial denaturation at 95 °C for 15 s followed by 30 cycles of 15 s at 95 °C, 15 s at 57 °C and 1min at 72 °C with a final extension step of 5 min at 72 °C.
TEF-1α (OP330068)	EF-1: ATG GGT AAG GAA GA CAA GAC<br>EF-2: GGA AGT ACC AGT GAT CAT GTT	Started with an initial denaturation at 95 °C for 5 min, followed by 30 cycles of 45 s at 94 °C, 40 s at 58 °C, and 1 min at 72 °C with a final extension of 10 min at 72 °C.
TUB (OP382711)	TUB-2Fd: GTB CAC CTY CAR ACC GGY CAR TG<br>TUB4RD: CCR GAY TGR CCR AAR ACR AAG TTG TC	Started with an initial denaturation at 94 °C for 5 min, followed by 30 cycles of 30 s at 94 °C, 30 s at 52 °C and 30 s at 72 °C with a final elongation of 7 min at 72 °C.

TABLE S2 Primers used in this study

No.	Primer	Primer Sequences（5'to 3'）	Remark
1	Gtr1_5F	ATGGCCTCCTACATCGACACCTC	PCR primers for amplification of *gtr1* gene
2	Gtr1_3R	TTAGGCGGCAGCAGTGGYGGTGTTG	
3	Gtr2_5F	ATGTCACTCAGTCCTCCAATTTACAATCTTGC	PCR primers for amplification of *gtr2* gene
4	Gtr2_3R	TTACTGCTGCCGGGGCTTAGTGATG	
5	Sch9_5F	ATGCATGGGGTTATTAACAAAAACGTCAGGTC	PCR primers for amplification of *sch9* gene
6	Sch9_3R	TTATACGTCAAAGTGAGAACCCCCCAC	
7	Tap42_5F	ATGTCTTCAGAAGAACCCCAATCTC	PCR primers for amplification of *tap42* gene
8	Tap42_3R	TCAACCTCTATTGAGAGTATTTCCTGC	
9	HYG_5F	ATGAAAAAGCCTGAACTCACCGCGAC	PCR primers for amplification of *hyg* gene
10	HYG_3R	CTATTCCTTTGCCCTCGGACGAGTGCTG	
11	Gtr1_A1	ATGGCCTCCTACATCGACAC	PCR primers for amplification of the upstream sequence of the *gtr1* gene for construction of the gene deletion vector
12	Gtr1_A2	GTCGTGACTGGGAAAACCCTGGCGAGGATGTAGATCTTTGCGGC	
13	Gtr1_A3	TCCTGTGTGAAATTGTTATCCGCTGTTGTGCGACAGAAGACCTT	PCR primers for amplification of the downstream sequence of the *gtr1* gene for construction of the gene deletion vector
14	Gtr1_A4	GGCGATCTGACAATTGAGCA	
15	Gtr2_A1	AGATGATACATCTGCCAGCG	PCR primers for amplification of the upstream sequence of the *gtr2* gene for construction
16	Gtr2_A2	GTCGTGACTGGGAAAACCCTGGCGAAGGATCGTGGTATTGAGGC	

			of the gene deletion vector
17	Gtr2_A3	TCCTGTGTGAAATTGTTATCCGCTCCTCTCAGATGACTACAAGCTC	PCR primers for amplification of the downstream sequence of the *gtr2* gene for construction of the gene deletion vector
18	Gtr2_A4	CTATCGTACGAGTCCTCCTTC	
19	HY	GGATGCCTCCGCTCGAAGTA	PCR primers for identification of *gtr1, gtr2* and *tap42* deletion mutant strain
20	YG	CGTTGCAAGACCTGCCTGAA	
21	Sch9_A1	ATCCGCGGACCGAATTTCAGTTGTGCCC	PCR primers for amplification of the upstream sequence of the *sch9* gene for construction of the gene deletion vector
22	Sch9_A2	TAGGATCCCGTCCTCATCGATGTGGTGA	
23	Sch9_A3	ATGTCGACCATCGCCGAACTGATCTTGG	PCR primers for amplification of the downstream sequence of the *sch9* gene for construction of the gene deletion vector
24	Sch9_A4	TTGGTACCCTCATCCGGTTTGCCTTCCT	
25	Sch9_A5	GACCAGTCATGACGGTGCTGAAGTCAG	PCR primers for identification of *sch9* deletion mutant strain
26	Sch9_A6	CGATATGTCCGTTAGCATCCAAGAGG	
27	Tap42_A1	TCACTAGTTCAGATGAGGATATGGCTGACC	PCR primers for amplification of the upstream sequence of the *tap42* gene for construction of the gene deletion vector
28	Tap42_A2	TAGGATCCGCAGAGATTGGGGTTCTTCT	
29	Tap42_A3	TAGTCGACCTTGGTTCACGGGATCAAATGTC	PCR primers for amplification of the downstream sequence of the *tap42* gene for construction of the gene deletion vector
30	Tap42_A4	ATGGTACCGCTTCGATGACCCAAGATAGACTC	
31	Tap42_A5	GTTGAAGCGAGCTCCAGAAGCGTGC	PCR primers for identification of *tap42* deletion mutant strain
32	Tap42_A6	GTCTCCTCTCTTCCTCGAGATACTCATCG	
33	Gtr1_C1	GAGAACGGTGAGAAAGCTAAGG	PCR primers for amplification of the full length of the *gtr1* gene and 5 'and 3' untranslated regions
34	Gtr1_C2	AACATGGCAGACCACGAAGCCCAGATCGTG	
35	Gtr2_C1	TGCAGAAATCCCTATCAGCTAG	PCR primers for amplification of the full length of the *gtr2* gene and 5 'and 3' untranslated regions
36	Gtr2_C2	CTTCATCTCATCCCACCCTATC	
37	Sch9_C1	GACTTCATCCTACCATCGACTC	PCR primers for amplification of the full length of the *sch9* gene and 5 'and 3' untranslated regions
38	Sch9_C2	AACGTCCAGACATTCAACGTCCCCAAG	

39	Tap42_C1	CAAGGATGACGACGAGTATGAG	PCR primers for amplification of the full length of the *tap42* gene and 5 'and 3' untranslated regions
40	Tap42_C2	CACGAATGAAGATGCGTACGAGACACC	
41	Gtr1-infu-5F	AGGTACCCGGGGATCCGAGAACGGTGAGAAAGCTAAGGC	PCR primers for amplification of the full length of the *gtr1* gene for construction of the gene complemented vector
42	Gtr1-infu-3R	GGCCAGTGCCAAGCTTAACATGGCAGACCACGAAGC	
43	Tap42-infu-5F	AGGTACCCGGGGATCCCAAGGATGACGACGAGTATGAGC	PCR primers for amplification of the full length of the *tap42* gene for construction of the gene complemented vector
44	Tap42-infu-3R	GGCCAGTGCCAAGCTTCACGAATGAAGATGCGTACG	
45	Sch9-infu-5F	AGGTACCCGGGGATCCGACTTCATCCTACCATCGACTCG	PCR primers for amplification of the full length of the *sch9* gene for construction of the gene complemented vector
46	Sch9-infu-3R	AGCTCTGCAGGTCGACAACGTCCAGACATTCAACGTCC	
47	Gtr2-infu-5F	AGGTACCCGGGGATCCTGCAGAAATCCCTATCAGCTAGG	PCR primers for amplification of the full length of the *gtr2* gene for construction of the gene complemented vector
48	Gtr2-infu-3R	AGCTCTGCAGGTCGACCTTCATCTCATCCCACCCTATCAAC	
49	neo-F	ATCTCGAGGGAGGTCAACACATCAATGCT	PCR primers for amplification of the *neo* gene for identification of complemented strain
50	neo-R	ATGGTACCTCAGAAGAACTCGTCAAGAAG	

Supplemental – Figures

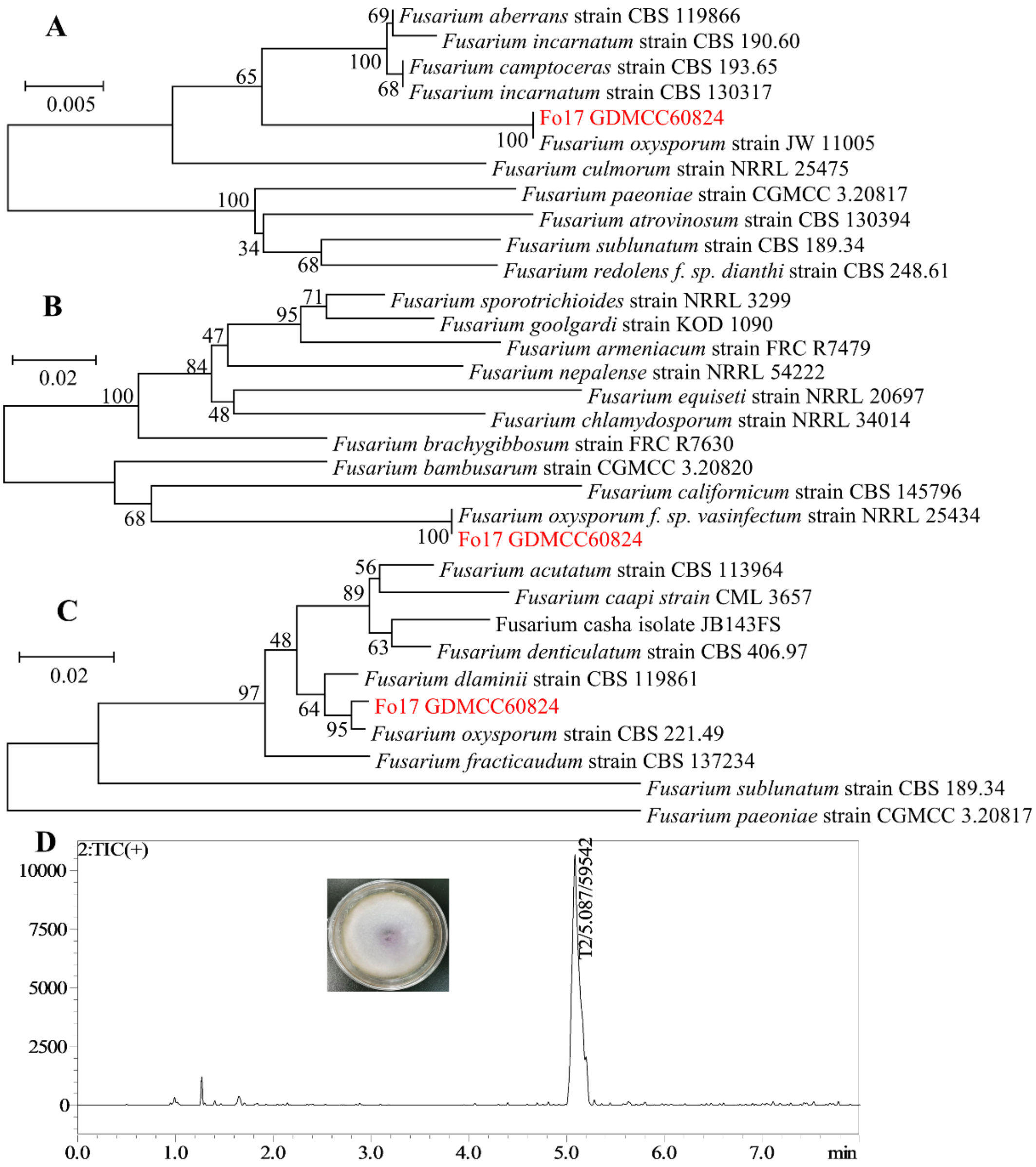

Figure S1. Neighbor-joining tree constructed based on the internal transcribed spacer (OP412776, A), translation elongation factor 1-alpha (OP330068, B) and β-tubulin (OP382711, C) sequences. Total ions chromatogram for T-2 toxin produced by *Fusarium oxysporum* (Fo17, GDMCC60824) (D).

```
1       AGTCAAGAAG CCCAAGAAGA AAAAGGTGCT CCTCATGGGA AAGTCCGGCT CCGGCAAGTC
61      AAGCATGAGG AGTATTATCT TTAGCAACTA CATTGCCCGC GATACACGAA GACTGTAAGT
121     CCCCCCTTGC CTAGGTATGG GGGTTGAAGC TGATTATGTA GGGGCGCAAC TATCGATATC
181     GATCTTTCGC ACGTCAAGTT CCTCGGAAAC CTCACCCTCA ATCTCTGGGA CTGCGGTGGT
241     CAAGAGGCCT TTATGGAGAA CTATCTCTCG CAACAGCGCG TCCACGTCTT TTCGAACGTC
301     GGCGTGCTAA TCTACGTCTT CGACATTGAA TCCCGCGACG TCGACCGCGA CTTGGCAACC
361     TACGTCTCCA TCCTCTCCGC GCTCCTGCAA TACTCCCCCG CCGCAAAGAT CTACATCCTC
421     GCCAGGGTTT TCCCAGTCAC GACTTGATTG TGTAAAACGA CGGCCAGTGT CTGAGGCTCG
481     CTGCAGACCT GAAGCTTGAT ATCGAATTCG CGTGTCGCCC TTATGAAAAA GCCTGAACTC
541     ACCGCGACGT CTGTCGAGAA GTTTCTGATC GAAAAGTTCG ACAGCGTCTC CGACCTGATG
601     CAGCTCTCGG AGGGCGAAGA ATCTCGTGCT TTCAGCTTCG ATGTAGGAGG GCGTGGATAT
661     GTCCTGCGGG TAAATAGCTG CGCCGATGGT TTCTACAAAG ATCGTTATGT TTATCGGCAC
721     TTTGCATCGG CCGCGCTCCC GATTCCGGAA GTGCTTGACA TTGGGGAGTT TAGCGAGAGC
781     CTGACCTATT GCATCTCCCG CCGTTCACAG GGTGTCACGT TGCAAGACCT GCCTGAAACC
841     GAACTGCCCG CTGTTCTACA ACCGGTCGCG GAGGCTATGG ATGCGATCGC TGCGGCCGAT
901     CTTAGCCAGA CGAGCGGGTT CGGCCCATTC GGACCGCAAG GAATCGGTCA ATACACTACA
961     TGGCGTGATT TCATATGCGC GATTGCTGAT CCCCATGTGT ATCACTGGCA AACTGTGATG
1021    GACGACACCG TCAGTGCGTC CGTCGCGCAG GCTCTCGATG AGCTGATGCT TTGGGCCGAG
1081    GACTGCCCCG AAGTCCGGCA CCTCGTGCAC GCGGATTTCG GCTCCAACAA TGTCCTGACG
1141    GACAATGGCC GCATAACAGC GGTCATTGAC TGGAGCGAGG CGATGTTCGG GGATTCCCAA
1201    TACGAGGTCG CCAACATCTT CTTCTGGAGG CCGTG
```

Figure S2. GTR1 knockout transformant AP (Gtr1-A1HY) fragment sequence

```
1       CCGGTCGCGG AGGCTATGGA TGCGATCGCT GCGGCCGATC TTAGCCAGAC GAGCGGGTTC
61      GGCCCATTCG GACCGCAAGG AATCGGTCAA TACACTACAT GGCGTGATTT CATATGCGCG
121     ATTGCTGATC CCCATGTGTA TCACTGGCAA ACTGTGATGG ACGACACCGT CAGTGCGTCC
181     GTCGCGCAGG CTCTCGATGA GCTGATGCTT TGGGCCGAGG ACTGCCCCGA AGTCCGGCAC
241     CTCGTGCACG CGGATTTCGG CTCCAACAAT GTCCTGACGG ACAATGGCCG CATAACAGCG
301     GTCATTGACT GGAGCGAGGC GATGTTCGGG GATTCCCAAT ACGAGGTCGC CAACATCTTC
361     TTCTGGAGGC CGTGGTTGGC TTGTATGGAG CAGCAGACGC GCTACTTCGA GCGGAGGCAT
421     CCGGAGCTTG CAGGATCGCC ACGACTCCGG GCGTATATGC TCCGCATTGG TCTTGACCAA
481     CTCTATCAGA GCTTGGTTGA CGGCAATTTC GATGATGCAG CTTGGGCGCA GGGTCGATGC
541     GACGCAATCG TCCGATCCGG AGCCGGGACT GTCGGGCGTA CACAAATCGC CCGCAGAAGC
601     GCGGCCGTCT GGACCGATGG CTGTGTAGAA GTACTCGCCG ATAGTGGAAA CCGACGCCCC
661     AGCACTCGTC CGAGGGCAAA GGAATGAAGG GCGACACGCG AATTCGATAT CAAGCTTCAG
721     GTCTGCAGTC AATACTGACG ATGGTCATAG CTGTTTCCTG TGTGAAATTG TTATCCGCTG
781     TTGTGCGACA GAAGACCTTC GAGTATGCGA ATTCAGTCGG TATTGCTGCG TCGTCGATTG
841     AGCTCACGCC GTTTGCGACG TCGATTTGGG ATCAGAGCTT GTATAAAGCG TGGGCGTCGA
901     TTATTCATGA CCTTGTGCCT AACCTCTCGG TGATTGAGCG AAATCTCGCA AACCTGGGTC
961     TCGCCATCGA AGCTGAAGAA TTGCTGCTGT TCGAGCGCAC ATCGTTCCTT GCCGTATCAT
1021    CCTGGACTTC GTCTGAAGGA CAACGAAACC CGACTGAGGA TCGACTAGAG CGCATGTCAA
1081    ACATCATGAA GCATTTCAAG CAGAGTATTT CGCGCTTCAC CGGTACGCCG CGCAACGCCG
1141    AGCAGTTCAT TCGAATGGAG CACAAGGCCG GCAATCGATT TAGTCTGTTT ATTCTCAAGT
1201    TCACGACAAA CACGTATCTC ATGGTTGT
```

Figure S3. GTR1 knockout transformant BP (Gtr1-A4YG) fragment sequence

```
1       CTTTTCGAAC AGTCAATTAG CGGAGCTTCG GCATCTAAAA AGGATGCACA AAAACCTGCC
61      GCCCCCGGAC CCAATTCTGT TGGCGCAGCT CCTAAGGGGA AGCCGCGCTT ATTACTCATG
121     GGTCAACGAA GGTATGATAT ACGTGGGAGA AAAGAGCAGA CATCAGCTAA CAGTATGTCA
181     CAGGAGCGGA AAGTCATCCA TCTCGAGTGT AGTCTTCCAC AAGCTGCCCC CGAACGAGAC
241     TCTGTTCCTT GAATCAACGG CTAGAATTCA AAAGGACTCC ATGGCGTTAG TGCTAGCAAT
301     ACTCTGACCG TGATGACCAT AAACTCATAA TTGCATAGGT CATTCATGGA TTTCCAAGTC
361     TGGGACTTTC CAGGCCAGAT CGACGTCTTT GAGAACCCAG GATTTGATAT TGAGGCCATC
421     TTCAGCGAGA TTGGAGCCCT CATCTGGGTC ATCGACGCCC AGGATGACTA CCTCGAAGCC
481     GTCATGCGCC TCAATACCAC GATCCTTCGC CAGGGTTTTC CCAGTCACGA CTTGATTGTG
541     TAAAACGACG GCCAGTGTCT GAGGCTCGCT GCAGACCTGA AGCTTGATAT CGAATTCGCG
601     TGTCGCCCTT ATGAAAAAGC CTGAACTCAC CGCGACGTCT GTCGAGAAGT TTCTGATCGA
661     AAAGTTCGAC AGCGTCTCCG ACCTGATGCA GCTCTCGGAG GGCGAAGAAT CTCGTGCTTT
721     CAGCTTCGAT GTAGGAGGGC GTGGATATGT CCTGCGGGTA AATAGCTGCG CCGATGGTTT
781     CTACAAAGAT CGTTATGTTT ATCGGCACTT TGCATCGGCC GCGCTCCCGA TTCCGGAAGT
841     GCTTGACATT GGGGAGTTTA GCGAGAGCCT GACCTATTGC ATCTCCCGCC GTTCACAGGG
901     TGTCACGTTG CAAGACCTGC CTGAAACCGA ACTGCCCGCT GTTCTACAAC CGGTCGCGGA
961     GGCTATGGAT GCGATCGCTG CGGCCGATCT TAGCCAGACG AGCGGGTTCG GCCCATTCGG
1021    ACCGCAAGGA ATCGGTCAAT ACACTACATG GCGTGATTTC ATATGCGCGA TTGCTGATCC
1081    CCATGTGTAT CACTGGCAAA CTGTGATGGA CGACACCGTC AGTGCGTCCG TCGCGCAGGC
1141    TCTCGATGAG CTGATGCTTT GGGCCGAGGA CTGCCCCGAA GTCCGGCACC TCGTGCACGC
1201    GGATTTCGGC TCCAACAATG TCCTGACGGA CAATGGCCGC ATAACAGCGG TCATTGACTG
1261    GAGCGAGGCG ATGTTCGGGG ATTCCCAATA CGAGGTCGCC AACATCTTCT TCTGGAGGCC
1321    GTG
```

Figure S4. GTR2 knockout transformant AP (Gtr2-A1HY) fragment sequence

```
1       GGAGGCTATG GATGCGATCG CTGCGGCCGA TCTTAGCCAG ACGAGCGGGT TCGGCCCATT
61      CGGACCGCAA GGAATCGGTC AATACACTAC ATGGCGTGAT TTCATATGCG CGATTGCTGA
121     TCCCCATGTG TATCACTGGC AAACTGTGAT GGACGACACC GTCAGTGCGT CCGTCGCGCA
181     GGCTCTCGAT GAGCTGATGC TTTGGGCCGA GGACTGCCCC GAAGTCCGGC ACCTCGTGCA
241     CGCGGATTTC GGCTCCAACA ATGTCCTGAC GGACAATGGC CGCATAACAG CGGTCATTGA
301     CTGGAGCGAG GCGATGTTCG GGGATTCCCA ATACGAGGTC GCCAACATCT TCTTCTGGAG
361     GCCGTGGTTG GCTTGTATGG AGCAGCAGAC GCGCTACTTC GAGCGGAGGC ATCCGGAGCT
421     TGCAGGATCG CCACGACTCC GGGCGTATAT GCTCCGCATT GGTCTTGACC AACTCTATCA
481     GAGCTTGGTT GACGGCAATT TCGATGATGC AGCTTGGGCG CAGGGTCGAT GCGACGCAAT
541     CGTCCGATCC GGAGCCGGGA CTGTCGGGCG TACACAAATC GCCCGCAGAA GCGCGGCCGT
601     CTGGACCGAT GGCTGTGTAG AAGTACTCGC CGATAGTGGA AACCGACGCC CCAGCACTCG
661     TCCGAGGGCA AAGGAATGAA GGGCGACACG CGAATTCGAT ATCAAGCTTC AGGTCTGCAG
721     TCAATACTGA CGATGGTCAT AGCTGTTTCC TGTGTGAAAT TGTTATCCGC TCCTCTCAGA
781     TGACTACAAG CTCGATATTC AGCGCGACAT CACCATCCGT ATTCAAGATG AGTTGTCAGA
841     CCACGGCTTT GAGAACGCCC CCGTCACCTT CCACCTAACC TCCATTTACA ACCATTCTAT
901     TTTCGAGGCT TTCAGCAAGG TTATCCAAAA GCTCATTCCT CGCCTTGGAA CGCTGGAATC
961     CATGCTTACC AACCTCTGCC GCACGTGCCG TTTCGAAAAG GCATACCTCT TTGATGTGCT
1021    TACAAAGATC TATATCGCCA CTGACAGCGC AACTGCCGAT ATGGCGAGCT ACGAGATCTG
1081    CAGTGATTAC ATCGATGTCA TCATTGATAT AACTGAGGTT TATGGTACGT GGCAGCGCAG
1141    TGATGAAGGG CGGCGTCGGC TGGAGGGTGA ACCGTGGAGC GCGCCTATCG ATAAACAGAT
1201    TGGCTGCAAC ACTGCAGAAA GCTGTCTTGT CCTGCACGAC GGAAACAAGC CCATCATGCT
1261    GCGTGAGGTG ATCGTTACCT TA
```

Figure S5. GTR2 knockout transformant BP (Gtr2-A4YG) fragment sequence

```
1       CGGGTACCTC AGAGTCTGCC GGTGTCCAGG CGCCTGCCGC CAAGGGAAAG CTAACAATCA
61      AGATCAATGA GGCTCGTGGG CTTAGGAAGA GTCGGGACCC TTATGTTGTT GTGGTTTTTC
121     AGCGTAGTGA GCTCATTTCT GGGGGTCCTC ACCACATCGA TGAGGACGGG ATCCTGGAGG
181     TCAACACTGA ATGCCTATTT TGGTTTAGTC GTCCAGGCGG TGAGCACAAA ATTTGTGTCG
241     TTTGACAAGA TGGTTCATTT AGGCAACTGG TCAGATCAGC CCCACTTGTA GCAGTAGCGG
301     CGGCGCTCGA AGTGTGACTC TTATTAGCAG ACAGGAACGA GGACATTATT ATCATCTGCT
361     GCTTGGTGCA CGATAACTTG GTGCGTTTGT CAAGCAAGGT AAGTGGACGA CCCGGTCATA
421     CCTTCTTAAG TTCGCCCTTC CTCCCTTTAT TTCAGATTCA ATCTGACTTA CCTATTCTAC
481     CCAAGCATCC AAATGAAAAA GCCTGAACTC ACCGCGACGT CTGTCGAGAA GTTTCTGATC
541     GAAAAGTTCG ACAGCGTCTC CGACCTGATG CAGCTCTCGG AGGGCGAAGA ATCTCGTGCT
601     TTCAGCTTCG ATGTAGGAGG GCGTGGATAT GTCCTGCGGG TAAATAGCTG CGCCGATGGT
661     TTCTACAAAG ATCGTTATGT TTATCGGCAC TTTGCATCGG CCGCGCTCCC GATTCCGGAA
721     GTGCTTGACA TTGGGGAGTT CAGCGAGAGC CTGACCTATT GCATCTCCCG CCGTGCACAG
781     GGTGTCACGT TGCAAGACCT GCCTGAAACC GAACTGCCCG CTGTTCTCCA GCCGGTCGCG
841     GAGGCCATGG ATGCGATCGC TGCGGCCGAT CTTAGCCAGA CGAGCGGGTT CGGCCCATTC
901     GGACCGCAAG GAATCGGTCA ATACACTACA TGGCGTGATT TCATATGCGC GATTGCTGAT
961     CCCCATGTGT ATCACTGGCA AACTGTGATG GACGACACCG TCAGTGCGTC CGTCGCGCAG
1021    GCTCTCGATG AGCTGATGCT TTGGGCCGAG GACTGCCCCG AAGTCCGGCA CCTCGTGCAT
1081    GCGGATTTCG GCTCCAACAA TGTCCTGACG GACAATGGCC GCATAACAGC GGTCATTGAC
1141    TGGAGCGGGG CGATGTTCGG GGATTCCCAA TACGAGGTCG CCAACATCCT CTTCTGGAGG
1201    CCGTGGTTGG CTTGTATGGA GCAGCAGACG CGCTACTTCG AGCGGAGGCA TCCGGAGCTT
1261    GCAGGATCGC CGCGCCTCCG GGCGTATATG CTCCGCATTG GTCTTGACCA ACTCTATCAG
1321    AGCTTGGTTG ACGGCAATTT CGATGATGCA GCTTGGGCGC AGGGTCGATG CGACGCAATC
1381    GTCCGATCCG GAGCCGGGAC TGTCGGGCGT ACACAAATCG CCCGCAGAAG CGCGGCCGTC
1441    TGGACCGATG GCTGTGTAGA AGTACTCGCC GATAGTGGAA ACCGACGCCC CAGCACTCGT
1501    CCGAGGGCAA AGGAATAGAG TAGGTCGACC ATCGCCGAAC TGATCTTGGC CATTCAGCAC
1561    CTTCATAATA ACGACATGTA TACCGATCTA GCAAA
```

Figure S6. Sch9 knockout transformant SP (Sch9-A5A6) fragment sequence

```
1       GACTGTAGTA GCGCGGGGTT CTCATCAAGA TCCAAGGGTC TCGTCTAGAT TTATCATCCC
61      CTACATTCGC CCCTCGTTAT TTACTTCAAT AAACTTCACA TCACAGCAAA TTATATTTAT
121     CATGTCTTCA GAAGAACCCC AATCTCTGCG GATCCTGGAG GTCAACACTG AATGCCTATT
181     TTGGTTTAGT CGTCCAGGCG GTGAGCACAA AATTTGTGTC GTTTGACAAG ATGGTTCATT
241     TAGGCAACTG GTCAGATCAG CCCCACTTGT AGCAGTAGCG GCGGCGCTCG AAGTGTGACT
301     CTTATTAGCA GACAGGAACG AGGACATTAT TATCATCTGC TGCTTGGTGC ACGATAACTT
361     GGTGCGTTTG TCAAGCAAGG TAAGTGGACG ACCCGGTCAT ACCTTCTTAA GTTCGCCCTT
421     CCTCCCTTTA TTTCAGATTC AATCTGACTT ACCTATTCTA CCCAAGCATC CAAATGAAAA
481     AGCCTGAACT CACCGCGACG TCTGTCGAGA AGTTTCTGAT CGAAAAGTTC GACAGCGTCT
541     CCGACCTGAT GCAGCTCTCG GAGGGCGAAG AATCTCGTGC TTTCAGCTTC GATGTAGGAG
601     GGCGTGGATA TGTCCTGCGG GTAAATAGCT GCGCCGATGG TTTCTACAAA GATCGTTATG
661     TTTATCGGCA CTTTGCATCG GCCGCGCTCC CGATTCCGGA AGTGCTTGAC ATTGGGGAGT
721     TCAGCGAGAG CCTGACCTAT TGCATCTCCC GCCGTGCACA GGGTGTCACG TTGCAAGACC
781     TGCCTGAAAC CGAACTGCCC GCTGTTCTCC AGCCGGTCGC GGAGGCCATG GATGCGATCG
841     CTGCGGCCGA TCTTAGCCAG ACGAGCGGGT TCGGCCCATT CGGACCGCAA GGAATCGGTC
901     AATACACTAC ATGGCGTGAT TTCATATGCG CGATTGCTGA TCCCCATGTG TATCACTGGC
961     AAACTGTGAT GGACGACACC GTCAGTGCGT CCGTCGCGCA GGCTCTCGAT GAGCTGATGC
1021    TTTGGGCCGA GGACTGCCCC GAAGTCCGGC ACCTCGTGCA TGCGGATTTC GGCTCCAACA
1081    ATGTCCTGAC GGACAATGGC CGCATAACAG CGGTCATTGA CTGGAGCGGG GCGATGTTCG
1141    GGGATTCCCA ATACGAGGTC GCCAACATCC TC
```

Figure S7. Tap42 knockout transformant AP (Tap42-A5HY) fragment sequence

```
1       AAGACCTGCC TGAAACCGAA CTGCCCGCTG TTCTCCAGCC GGTCGCGGAG GCCATGGATG
61      CGATCGCTGC GGCCGATCTT AGCCAGACGA GCGGGTTCGG CCCATTCGGA CCGCAAGGAA
121     TCGGTCAATA CACTACATGG CGTGATTTCA TATGCGCGAT TGCTGATCCC CATGTGTATC
181     ACTGGCAAAC TGTGATGGAC GACACCGTCA GTGCGTCCGT CGCGCAGGCT CTCGATGAGC
241     TGATGCTTTG GGCCGAGGAC TGCCCCGAAG TCCGGCACCT CGTGCATGCG GATTTCGGCT
301     CCAACAATGT CCTGACGGAC AATGGCCGCA TAACAGCGGT CATTGACTGG AGCGGGGCGA
361     TGTTCGGGGA TTCCCAATAC GAGGTCGCCA ACATCCTCTT CTGGAGGCCG TGGTTGGCTT
421     GTATGGAGCA GCAGACGCGC TACTTCGAGC GGAGGCATCC GGAGCTTGCA GGATCGCCGC
481     GCCTCCGGGC GTATATGCTC CGCATTGGTC TTGACCAACT CTATCAGAGC TTGGTTGACG
541     GCAATTTCGA TGATGCAGCT TGGGCGCAGG GTCGATGCGA CGCAATCGTC CGATCCGGAG
601     CCGGGACTGT CGGGCGTACA CAAATCGCCC GCAGAAGCGC GGCCGTCTGG ACCGATGGCT
661     GTGTAGAAGT ACTCGCCGAT AGTGGAAACC GACGCCCCAG CACTCGTCCG AGGGCAAAGG
721     AATAGAGTAG GTCGACCTTG GTTCACGGGA TCAAATGTCA CGAGATGTGT CGCTCAGGCA
781     CAACTGCCGT AACTTCCTT
```

Figure S8. Tap42 knockout transformant BP (Tap42-A6YG) fragment sequence
